# Pegylation Reduces the Uptake of Certolizumab Pegol by Dendritic Cells and Epitope Presentation to T-Cells

**DOI:** 10.3389/fimmu.2022.808606

**Published:** 2022-02-04

**Authors:** Marie de Bourayne, Sylvain Meunier, Samuel Bitoun, Evelyne Correia, Xavier Mariette, Hervé Nozach, Bernard Maillère

**Affiliations:** ^1^ Université de Paris-Saclay, CEA, INRAE, Département Médicaments et Technologies pour la Santé, SIMoS, Gif-sur-Yvette, France; ^2^ Université Paris-Saclay, Assistance Publique-Hôpitaux de Paris, Hôpital Bicêtre, Department of Rheumatology, INSERM UMR1184, Le Kremlin Bicêtre, France

**Keywords:** certolizumab pegol, immunogenicity, PEGylation, CD4 T-cell response, T-cell epitope

## Abstract

Pegylation of biopharmaceuticals is the most common strategy to increase their half-life in the blood and is associated with a reduced immunogenicity. As antigen presentation is a primary event in the activation of CD4 T-cells and initiation of Anti-Drug Antibody (ADA) response, we investigated the role of the PEG molecule on the T-cell reactivity of certolizumab pegol (CZP), a pegylated anti-TNFα Fab. We generated T-cell lines raised against CZP and its non-pegylated form (CZNP) and demonstrated CZP primed few T-cells in comparison to CZNP. CZP-primed lines from 3 donors responded to a total of 5 epitopes, while CZNP-primed lines from 3 donors responded to a total of 7 epitopes, 4 epitopes were recognized by both CZP- and CZNP-primed lines. In line with this difference of T-cell reactivity, CZP is less internalized by the dendritic cells than CZNP. *In vitro* digestion assay of CZP by Cathepsin B showed a rapid removal of the PEG moiety, suggesting a limited influence of PEG on CZP proteolysis. We therefore demonstrate that pegylation diminishes antigen capture by dendritic cells, peptide presentation to T-cells and T-cell priming. This mechanism might reduce immunogenicity and contribute to the long half-life of CZP and possibly of other pegylated molecules.

## Introduction

Introduction of biopharmaceuticals (BP) brought clear clinical benefits for patients suffering from chronic inflammatory diseases such as rheumatoid arthritis or Crohn’s disease (1). These BPs target multiple molecules such as TNFα, IL-6R, CD20 and CD80/86, TNFα antagonists being most frequently used in the clinic. Most of BPs are full-length antibodies, which are either chimeric (infliximab, rituximab), humanized (tocilizumab) or fully human (golimumab, adalimumab) antibodies. Two other molecules are fusion proteins composed of the extracellular domain of the TNF receptor (etanercept) or CTLA-4 (abatacept), fused to an IgG1 Fc domain. Certolizumab pegol (CZP) has a unique structure consisting of a single Fab of a humanized anti-TNF antibody conjugated with a branched PEG through a maleimide linker to a specific cysteine residue in the hinge region, the two branches being of about 20kDa (2). This large PEG moiety limits the glomerular filtration of CZP and hence increase its half-life in the blood, which is close to that of a full-length antibody (2). The PEG moiety also facilitates the solubility of the molecules at high concentrations, compatible with subcutaneous administration (3). Furthermore, the lack of Fc prevents binding of CZP to FcRn and transfer of CZP across the materno-foetal barrier (4, 5).

The diversity of drug structures and of validated targets in inflammatory diseases enlarges the choice for clinicians to select the most appropriate drug for the patient. However, a major caveat of BPs is the risk that they might induce Anti-Drug Antibody (ADA) response, which might alter the PK of the BPs, diminish their clinical efficacy or induce hypersensitivity reactions (6–8). ADAs against infliximab (9), rituximab (10) or adalimumab (11) generally affect more than 20% of the patients, while the ADA occurrence is below 10% for tocilizumab (12, 13) and golimumab (8). In contrast, both fusion proteins (abatacept and etanercept) only generated ADA responses in rare cases (14, 15). Immunogenicity incidence of Certolizumab pegol appears to be variable across studies depending on the sensitivity of the assays (16–18) but the ADA response was mainly characterized by low titers and by an effect on PK for highest titers, only (https://www.ema.europa.eu). In line with the clinical immunogenicity, CD4 T-cell epitopes have been identified in many of these antibodies (19–22) and underlined the role of mutations with respect to the germline sequences to drive the T-cell response (20–22). Evaluation of the size of the specific T-cell repertoire before injection of the BPs using healthy donors also showed a good concordance with clinical immunogenicity (13, 21, 23, 24). These findings are in agreement with previous observations that the size of the naïve repertoire contributes to the intensity of the T-cell response (25, 26). Owing to its unique structure, the root cause of immunogenicity of CZP might be different from other BPs, especially because of the presence of the branched PEG. Pegylation is generally associated with a low immunogenicity of the BPs (27) and has been suggested to mask epitopes to antibody recognition. However, antibodies to PEGs including preexisting antibodies (28) or induced by the pegylated products (29) have been reported, showing that PEGs bring additional antigenic determinants (30). In order to provide insights on the role of PEG for reducing immunogenicity, we compared the ability of certolizumab pegol (CZP) and non-pegylated certolizumab (CZNP) to prime CD4 T-cells and deciphered the influence of PEG on peptide presentation to T-cells.

## Material and Methods

### Proteins and Peptides

Adalimumab (Abbvie, North Chicago, Illinois), golimumab (Janssen, Beerse, Belgium) and abatacept (Bristol Myers Squibb, New York) were obtained from the pharmacy of Bicêtre hospital, AP-HP (Le Kremlin Bicetre, France). Certolizumab pegol (CZP) and non-pegylated Certolizumal (CZNP) were provided by UCB, Brussels, Belgium. Peptides were purchased from Pepscan (Lelystad, The Netherlands).

### Characterization of Protein-Specific CD4 T-Cell Lines

Peripheral blood mononuclear cells (PBMCs) were purified from the blood of anonymous healthy donors who gave informed consent (Etablissement Français du Sang, Rungis, France). Monocyte-derived dendritic cells (DCs) were generated from plastic-adherent cells of PBMCs after 5-day of culture (31), while CD4 T-cells were isolated from autologous non-adherent PBMCs using anti-CD4 immuno-magnetic beads (Miltenyi Biotech, Bergisch Gladbach, Germany) (31). DCs were loaded overnight at 37°C with either antibody or Fab (1 µM) or Keyhole Limpet Hemocyanin (KLH) (0.25 µM) (Thermo; Brebières, France) and matured with lipopolysaccharide (1 µg/mL). 2×10^5^ autologous CD4 T-cells per well were amplified by weekly rounds of stimulation with 2×10^4^ protein-loaded DCs and appropriate cocktails of cytokines during 21 days (31). Specificity of each T-cell line (expanded T-cell contained in one well) was assessed by IFN-γ ELISPOT (31). Briefly, multisScreen hemagglutinin 96-well plates (Merck Millipore, Fontenay sous Bois, France) were coated overnight at 4°C with 2.5 µg/mL anti-human IFN-γ mAb (1-D1K; Mabtech, Nacka Strand, Sweden) in PBS (Invitrogen). Wells were saturated for 2 h at 37°C with Iscove’s modified Dulbecco medium supplemented by 10% human AB serum (IMDM, Lonza, Levallois-Perret, France) and washed with PBS. Antibodies (3 µM) or KLH (1µM) was loaded onto iDCs in AIM-V for 4 h at 37°C, while peptides (10 µg/mL) were directly added to MultisScreen plates. PBMCs (5×10^4^/well) or iDCs (5×10^3^/well) were used as antigen-presenting cells and co-cultured in the plates with 30x10^3^ CD4 T-cells in AIM-V supplemented with 0.5 ng/mL rh-IL-7. After overnight incubation at 37°C and washing, plates were subsequently treated with 0.25 µg/mL biotinylated anti-human IFN-γ mAb (7-B6-1; Mabtech) in PBS/BSA 1%, extravidin-phosphatase (dilution 1:3000 in PBS/Tween 20 0.05%/BSA 1%; Sigma-Aldrich) and NBT/BCIP (Sigma-Aldrich). Spot number was determined by the AID ELISPOT Reader System (AID GmbH, Ebinger, Germany).CD4 T-cell lines were considered as specific when a spot count was 2-fold higher in the presence of the protein or the peptide than in their absence, with a minimal difference of 25 spots. The frequency of pre-existing CD4 T-cells was calculated by considering that the T-cell distribution follows the Poisson distribution, as previously described (23).

### Internalization of Labelled-Fabs by DCs

Both Fabs were labelled with a cyanine-5 by reaction on the ε-amino groups of their lysine residues of a ten-fold excess of sulfo N-hydro-succimidyl ester cyanine-5 with 1 mg/ml of each Fab. The final labelling was assessed by UV-visible spectrophotometric absorbance and was of 3.2 and 3.4 Cyanine-5 per Fab for CZP and CZNP, respectively. Immature DCs were produced from CD14+ cells purified from PBMCs with anti-CD14 immuno-magnetic beads (Miltenyi Biotech, Bergisch Gladbach, Germany) and incubated (100 000 cells/200µL) with each labelled Fab (2µM) in AIM V medium at varying times and directly introduced into a FACS Aria III cytometer (Becton Dickinson, Rungis, France) to assess Fab internalization.

### Proteolytic Degradation of Fabs

CZP and CZNP were incubated with Cathepsin B (R&D System, Minneapolis, Minnesota) or Cathepsin S (R&D System Minneapolis, Minnesota) with a 10:1 molar ratio (Fab 2µM: Cathepsin 0,2 µM) in the appropriate digestion buffer at varying times at 37° C. Cathepsin B digestion buffer: MES pH5 25mM, 50µM DTT. Cathepsin S digestion buffer: NaOAc 50 mM pH 4.5, 250mM NaCl, 50µM DTT. Cathepsin B and S were activated in the appropriate buffer with 1mM DTT before dilution and use. Reaction was stopped by addition of 5x Laemmli buffer and stored at 4°C before SDS-PAGE analysis. Degradation of Fab was assessed in SDS-PAGE after silver staining of the gels.

## Results

### CZP Induced Fewer T-Cells From Healthy Donors Than Other Anti-TNF Antagonists and the Non-Pegylated Form CZNP

**Figure 1:**
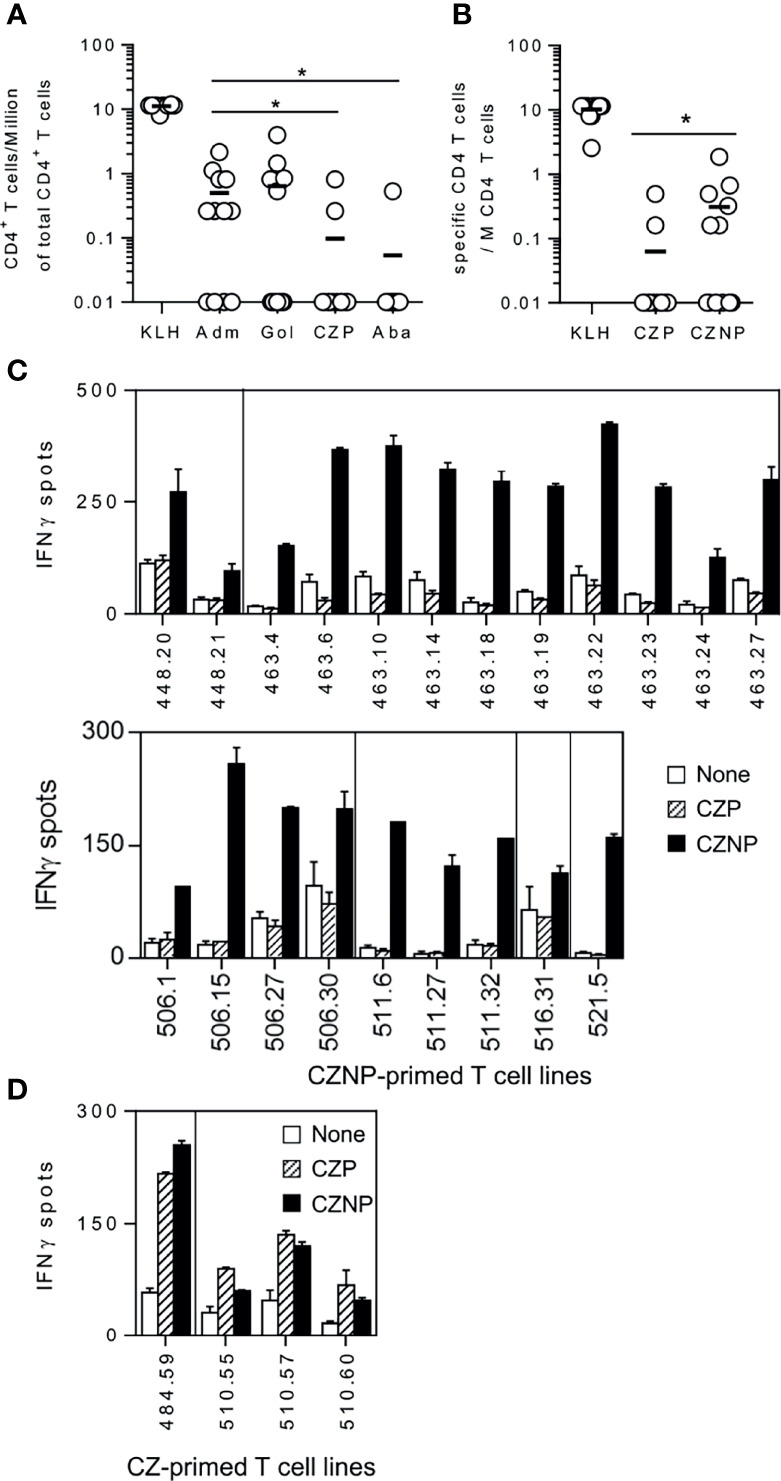
Comparison of the size of T-cell repertoire specific for therapeutic proteins in healthy donors. CD4 T-cell lines were generated by co-culturing CD4 T-cells with autologous DC loaded with each antibody. After 3 weeks, specificity of the T-cell lines were assessed by Elispot IFN-γ. CD4 T-cell lines were considered as specific when a spot count of duplicates was 2-fold higher in the presence of the protein (3µM) than in their absence, with a minimal difference of 25 spots. Frequencies of specific CD4 T-cells was estimated using the Poisson distribution according to the following formula: Frequency = -Ln((number of negative wells/total number of wells tested))/(number of CD4+ T-cells/well). **(A)** T-Cell frequencies evaluated with cells collected from 12 donors for Adalimumab, Golimumab, Certolizumab pegol and Abatacept. **(B)** comparison of T-cell frequencies between Certolizumab pegol (CZP) and its non-pegylated counterpart (CZNP). Specific T-cell lines raised against **(C)** CZNP **(D)** CZP were tested by Elispot with unloaded DCs (open bars), DCs loaded with CZP (hatched bars) and CZNP (black bars). Wilcoxon signed-rank test: *p < 0.05.

With the aim of explaining the differences of immunogenicity across BPs, we evaluated their capacity to prime CD4 T-cells collected from healthy donors. By using healthy donors, we mimicked the T-cell repertoire conditions at the first injection in the patients. To detect the protein-specific CD4 T-cells, CD4 T-cells collected from healthy donors were distributed into multiple wells and stimulated weekly with autologous DCs previously loaded with the different proteins. Specificity was assessed by IFN-γ ELISpot, the naïve T-cell being skewed to a Th1 phenotype by addition of IL-12 at the beginning of the cell culture. Because of the low frequency of antigen-specific naïve CD4 T-cells, only part of the T-cell lines (CD4 T-cells present in a single well) contained protein-specific CD4 T-cells, which are distributed upon the Poisson’s distribution at the initiation of the culture (**Supplemental Figure S1**). This distribution allowed us to calculate the frequency of specific CD4 T-cells for each of the proteins as demonstrated previously (23). All the 12 donors responded to KLH, demonstrating isolated cells are able to mount an *in vitro* T-cell response. Adalimumab and golimumab generated specific T-cell lines in 8 and 5 donors respectively, with a mean frequency of 0.5 and 0.6 CD4 T-cell per Million of CD4 T-cells (**Figure 1A**). In contrast, certolizumab pegol and abatacept gave rise to T-cell lines in very few donors with a lower mean frequency of 0.09 and 0.05 CD4 T-cell per Million, respectively. We then compared using another set of 12 healthy donors the priming ability of Certolizumab pegol (CZP) to its non-pegylated form (CZNP). Interestingly, many more T-cell lines (**Supplemental Figure S2**) were generated with CZNP (**Figure 1C**) as compared to CZP (**Figure 1D**), the mean frequency being of 0.3 and 0.06 CD4 T-cell per Million for CNZP and CZP, respectively (**Figure 1B**). Further, T-cells primed with CZNP did not recognize CZP, while a good cross reactivity was found between both molecules with T-cells primed with CZP, suggesting their fine specificity is different. Thus, CZP induced few T-cells as compared to other anti-TNFα antagonists, the PEG moiety contributing to a reduction of T-cell priming.

### CZP and CZNP Share Several CD4 T-Cell Epitopes

**Figure 2:**
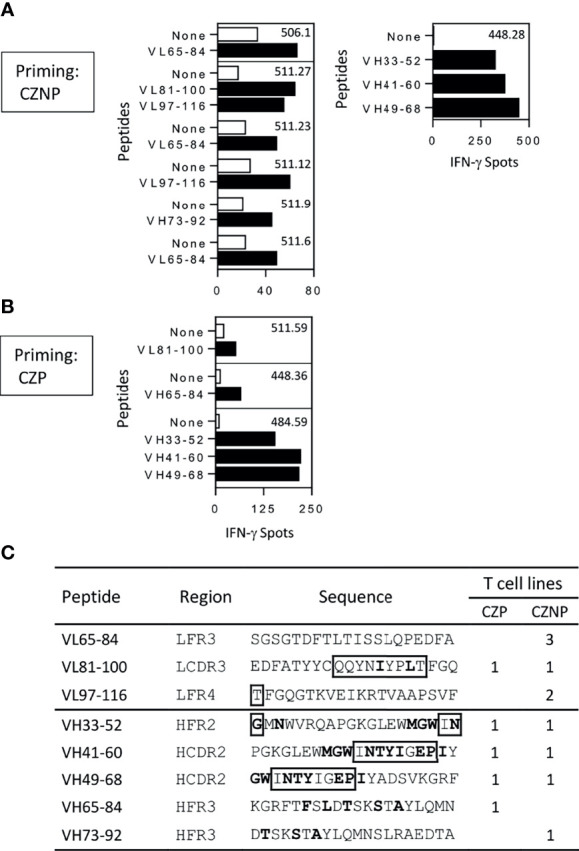
Mapping of T-cell epitopes of certolizumab pegol (CZP) and non-pegylated certolizumab (CZNP). T-cell lines generated from cells from PBMCs collected from 12 healthy donors and reacting with the peptide pools (**Figure S3**), were tested by Elispot with individual peptides (10µg/ml) (black bars) and without peptide (open bars). Only T cell lines reacting positively in the two independent Elispot assays were presented. T-cell lines were raised against **(A)** CZNP **(B)** CZP. **(C)** Summary of the mapping. Bold: Mutations with respect germline sequences. boxed residues: amino acids of CDR regions.

**Figure 3:**
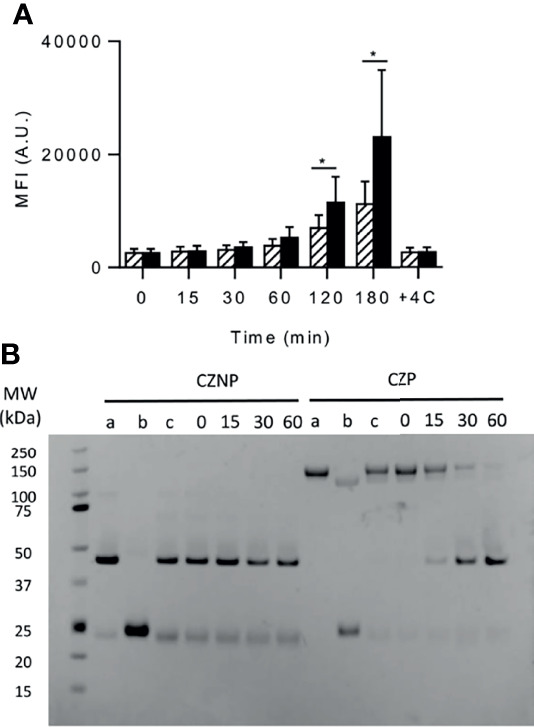
uptake by DCs and proteolytic degradation of certolizumab pegol (CZP) and non-pegylated certolizumab (CZNP). **(A)** Labelled CZP (2µM) (hatched bars) and CZNP (2µM) (black bars) were incubated with DCs (100 000 cells/200µL) and their uptake assessed by flow cytometry (Mean fluorescent Intensity (MFI). Data presented are the mean of 5 independent experiments carried out with cells produced from different donors **(B)** *In vitro* kinetic degradation of Fabs by Cathepsin **(B)** a: Fab. b: Fab + DTT. c: Fab without enzyme. Time is expressed in mn.

Peptide specificity of T-cell lines raised against either CZP or CZNP was investigated using overlapping 20-mer peptides encompassing the whole sequence of the VH and VL domains of CZP. T-cell lines were submitted to a first Elispot assay using peptide pools (**Supplemental Figure S3** and **Table S1**) and their specificity was confirmed in a second independent Elispot with individual peptides (**Figure 2**). As CZP gave rise to few T-cell lines, we identified CZP-specific T-cell epitopes from only 3 T-cell lines, one each from 3 donors, which were specific for 5 different peptides (**Figure 2A**). CZNP-specific T-cell epitopes were delineated from 7 different T-cell lines, also from 3 donors. As shown in **Figure 2C**, 3 and 4 epitopes were found in the VL and VH domains, respectively and mainly contained mutations with respect to the germline sequences. Four epitopes were recognized by both CZP- and CZNP-primed lines but we found only one donor, whose cells primed by either CZ or CZNP respond to a common peptide (VL81-100).

### The PEG Moiety Reduces the CZP Uptake by DCs

We assessed the uptake of both molecules by incubating them with immature dendritic cells and by evaluating their internalization by flow cytometry. As shown in **Figure 3A**, the cell fluorescence progressively increased along the time for both molecules but after 2 to 3 hours a significant higher uptake was observed for CZNP compared to CZP. Confocal microscopy confirmed the internalization of both molecule but did not reveal any differences of subcellular compartments reached (data not shown). We also evaluated the sensitivity to degradation of both molecules by endosomal proteases. While native CZP migrated at a MW over 100kD, its non-pegylated counterpart migrated at a MW close to 50kD (**Figure 3B**, lanes a). Reducing conditions (lanes b) separated the heavy and light chains but did not remove the PEG from the heavy chain. Under the tested conditions, cathepsin B did not cleave CZNP but removed the PEG moiety from CZP in the 30 mins of incubation (**Figure 3B**). Cathepsin S did not show any degradation of each of the molecules (data not shown). Probably because the PEG is fixed to the C-terminal of the heavy chain by a peptide of 8 AA, which might be flexible, it is rapidly removed from CZNP by cathepsin B. PEG should not therefore protect or delay the degradation of CZP in the endosomal compartment but was demonstrated to dampen internalization of CZP into DCs.

## Discussion

Certolizumab Pegol has a unique scaffold relying on a Fab format to which a large branched PEG is conjugated (2). We showed in this study that CZP induced fewer T-cells than the non-pegylated form CZNP, shared multiple epitopes with CZNP and was less captured by DC than CZNP. We therefore highlight that pegylation reduces presentation by DCs and activation of CZP-specific T-cells by a mechanism, which dampens its immunogenicity.

To decipher the mechanisms of CZP presentation to T-cells, we generated T-cell lines from cells collected from healthy donors with several BPs including CZP and CZNP. This approach allows us to obtain T-cells raised against both CZP and CZNP. Further, use of cells of healthy donors recapitulates the T-cell repertoire conditions at the first injection in the patients before any injection of the drug. In previous studies, immunogenic therapeutic proteins or antibodies exhibited a larger specific naïve T-cell repertoire in this assay than weakly immunogenic molecules (13, 23, 24). Accordingly, frequency of adalimumab- specific T-cells was found in this study in the range of 0.1 to 1 cell/Million of CD4 T-cell as all the immunogenic antibodies (23). In line with the frequency of pre-existing T cells, adalimumab is known to be immunogenic in approximately one third of rheumatoid arthritis patients (11). Golimumab exhibits a similar pre-existing T cell frequency, although its immunogenicity rate appears to be lower than adalimumab (8). In contrast, the weakly immunogenic fusion protein abatacept (15), exhibited a T-cell frequency below 0.1 cell/Million of CD4 T-cell as already observed for the other low immunogenic fusion protein etanercept (23). The size of the T-cell-repertoire specific for CZP was also reduced and might account for the low immunogenicity incidence of CZP observed in several studies (16–18) and the low titers observed in the more recent studies (https://www.ema.europa.eu). Interestingly, the absence of PEG made CZNP more able to prime T-cells than CZP did. The PEG moiety is conjugated to the Fab fragment on a cysteine residue, included on a small external peptide of 8 amino acids length, derived from the hinge sequence. In line with its location on a flexible peptide, the PEG moiety seems to be rapidly removed from the Fab structure as shown in the experiments of cathepsin degradation. CZ and CZNP did not induce DC maturation. Pegylation was associated with a weaker internalization by the DCs of CZP as compared to CZNP. This difference accounts for the difference of T-cell priming but also of T-cell cross-recognition between both molecules. Indeed, lack of recognition of CZP by CZNP-specific T-cells might result from its weak internalization in the Elispot assay, while the recognition of CZNP by CZP is facilitated by its uptake. PEG has been already shown to reduce uptake of nanoparticles by macrophages (32) and asialofetuin by hepatocytes (33). The molecular mechanism remains unclear but as no specific receptors are involved, we suggest that pegylation might interfere on the fluid phase uptake by reducing the local protein concentration and limiting interaction with cell surface components but this clearly remains a hypothesis. Pegylation was associated with a prolonged half-life of both types of compounds in the blood (32, 33). Pharmacokinetics of proteins in the body is known to be mainly governed by two mechanisms: the glomerular filtration in the kidneys and their uptake by phagocytes and endothelial cells leading to their degradation in the endosomes (34). Antibodies and serum albumin have a prolonged half-life thanks to their binding to FcRn in the cell endosomes, which prevents their intracellular degradation and allows their release into the blood stream. Their size also prevent their filtration by the kidney. The branched PEG moiety of CZP is large enough to avoid glomerular filtration but the lack of Fc domain and thus, absence of binding to FcRn (4) makes CZP susceptible to endosomal degradation. Limitation of the cell uptake provided by the PEGs might therefore contribute to increase the lifetime of CZP in the body in addition to reduce T-cell priming to CZP.

CZP is the only pegylated BP with an antibody-like structure but many BPs, including cytokines (IFN-α, IFN-β, GM-CSF) (35, 36) and replacement proteins (Factor VIII, lysosomal enzymes) (29, 37), have been empirically pegylated to increase their lifespan in the body. Pegylation was associated for many of these proteins with a lower immunogenicity level as compared to the non-pegylated forms (29, 35–37). Their conjugated PEGs vary in size, might be branched or in a single strand and might be unique or multiple per molecule. It remains unknown to what extent these attributes interfere with the cellular uptake (32) but it seems possible to speculate that the reduced uptake provided by pegylation that we observed for CZP, might be extended to other therapeutic proteins and might explain their overall reduced immunogenicity. We therefore highlighted in this paper that pegylation, which is used for decades to empirically increase half-life and reduce immunogenicity (27), might act, by mitigating antigen capture by phagocytes such as dendritic cells and T-cell priming raised against therapeutic proteins. Further studies including other pegylated biopharmaceuticals and different PEG linkages would be interesting to conduct and might generalize the observations we made with CZP. The demonstration of the mechanisms leading to lower immunogenicity of a pegylated Fab compared to its non-pegylated form may be useful for the development of new therapeutic antibodies.

## Data Availability Statement

The raw data supporting the conclusions of this article will be made available by the authors, without undue reservation.

## Ethics Statement

Ethical review and approval was not required for the study on human participants in accordance with the local legislation and institutional requirements. The patients/participants provided their written informed consent to participate in this study.

## Author Contributions

BM designed the experiments, analyzed the data and wrote the manuscript. MB, SM, EC, and HN designed and performed the experiments, analyzed the data and corrected the manuscript. SB and XM designed the experiments and corrected the manuscript. All authors contributed to the article and approved the submitted version.

## Conflict of Interest

The authors declare that the research was conducted in the absence of any commercial or financial relationships that could be construed as a potential conflict of interest.

## Publisher’s Note

All claims expressed in this article are solely those of the authors and do not necessarily represent those of their affiliated organizations, or those of the publisher, the editors and the reviewers. Any product that may be evaluated in this article, or claim that may be made by its manufacturer, is not guaranteed or endorsed by the publisher.
