# Supplementary figures and images for: Pegylation Reduces the Uptake of Certolizumab Pegol by Dendritic Cells and Epitope Presentation to T-Cells
Source: Front Immunol. 2022 Feb 4;13:808606. doi: 10.3389/fimmu.2022.808606 (PMC8854214; doi:10.3389/fimmu.2022.808606)

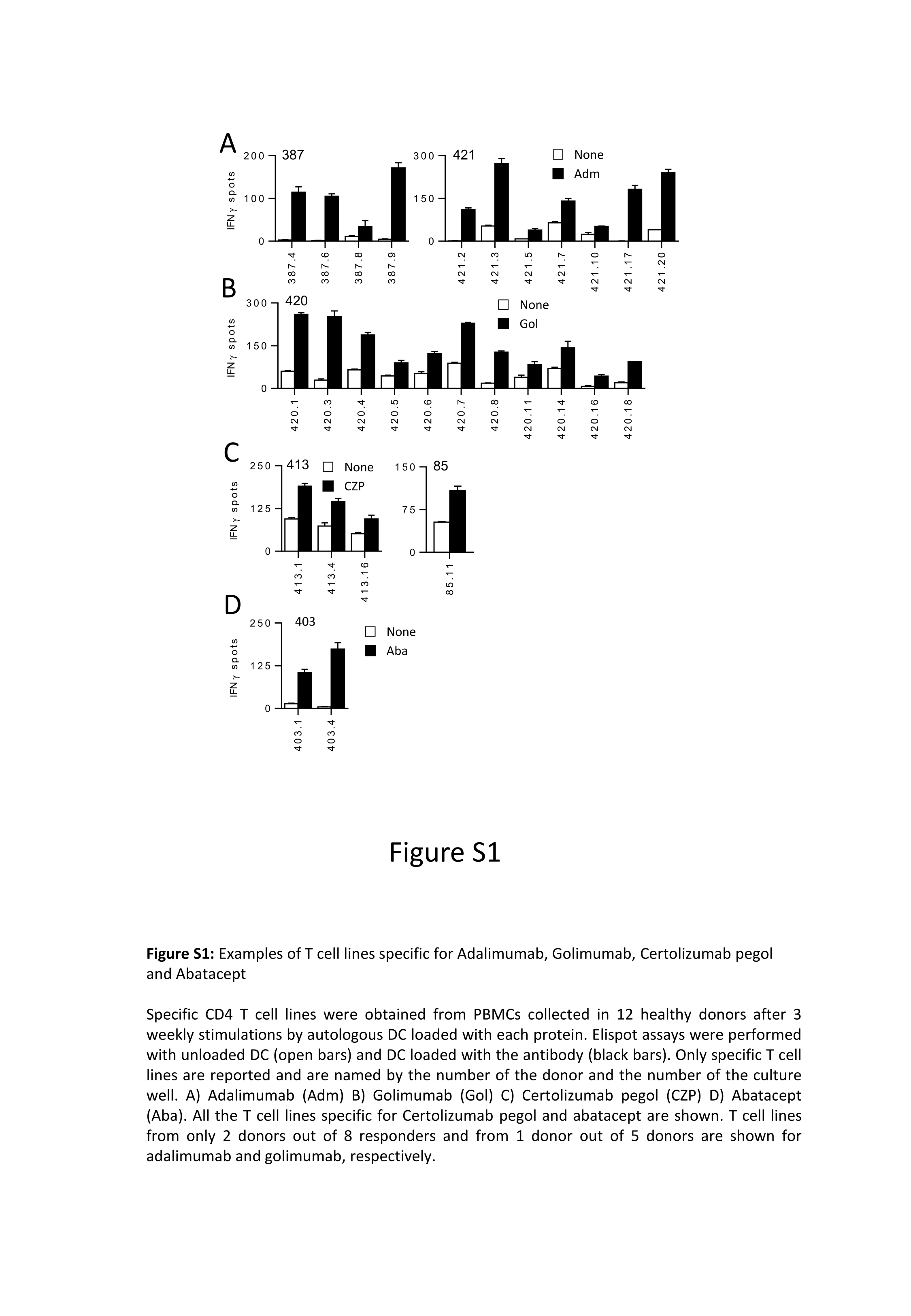

Supplement: Supplementary file 1 [file Image_1.tif]

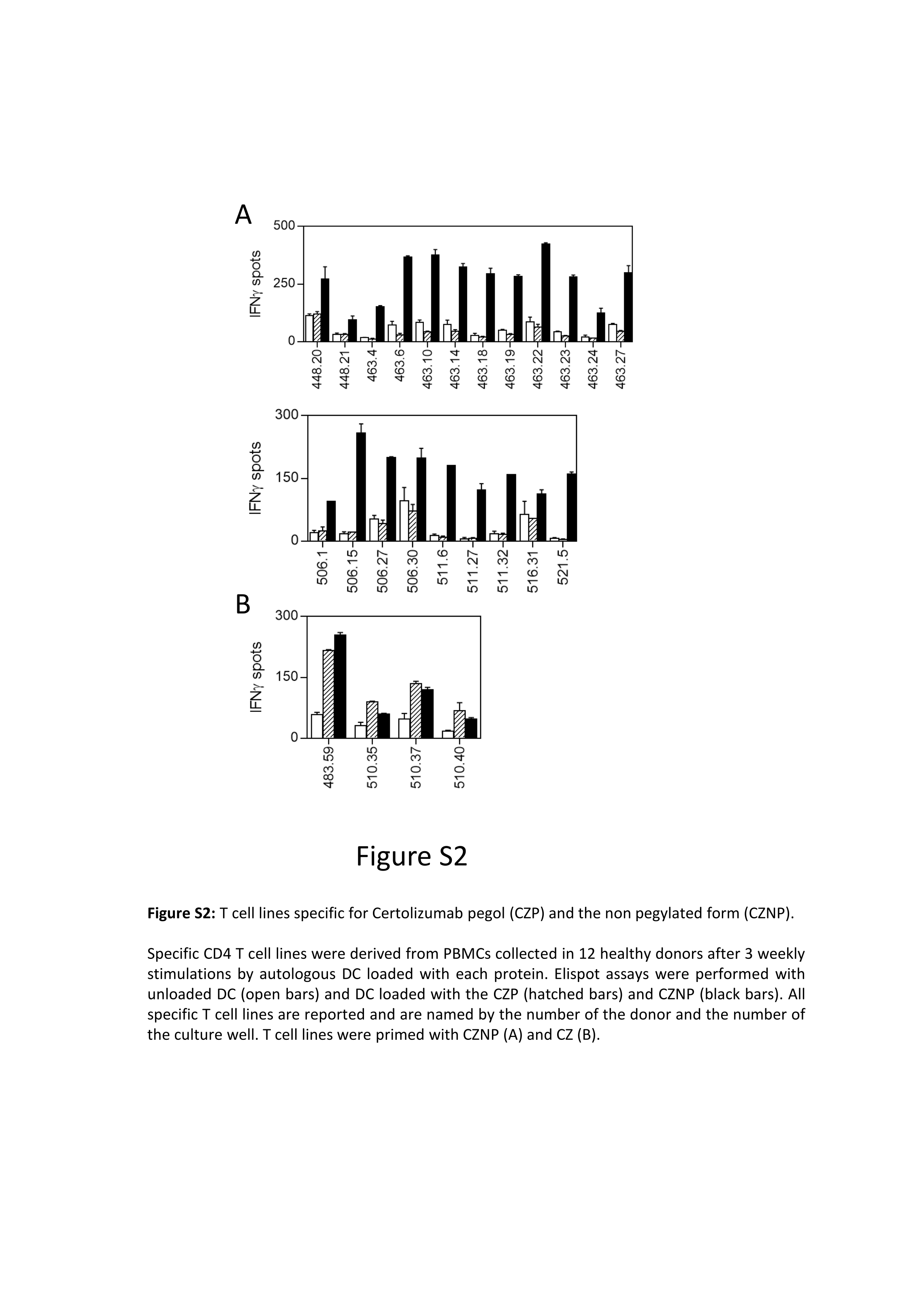

Supplement: Supplementary file 2 [file Image_2.tif]

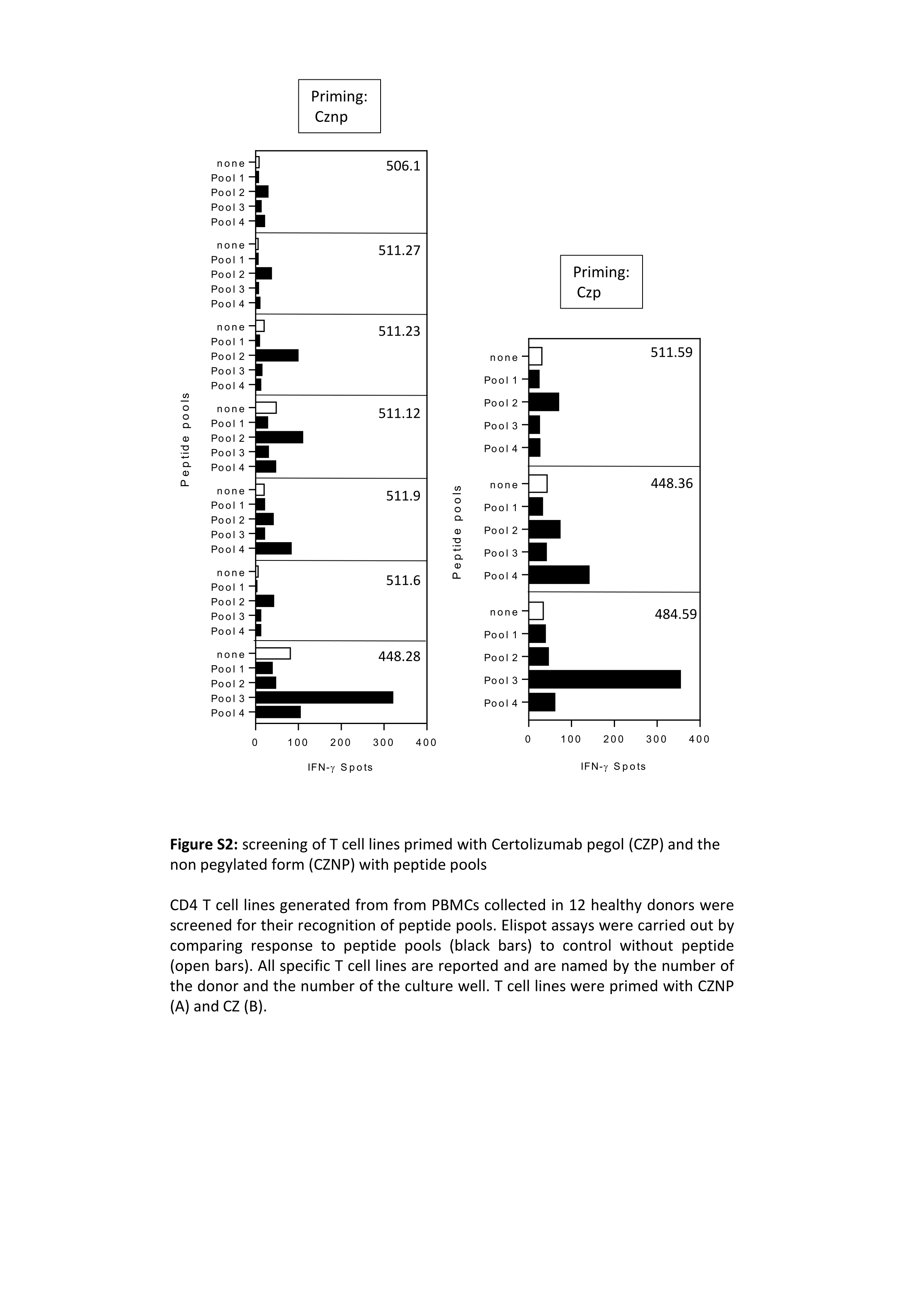

Supplement: Supplementary file 3 [file Image_3.tif]
