# Supplementary material for: Pegylation Reduces the Uptake of Certolizumab Pegol by Dendritic Cells and Epitope Presentation to T-Cells
Source: Front Immunol. 2022 Feb 4;13:808606. doi: 10.3389/fimmu.2022.808606 (PMC8854214; doi:10.3389/fimmu.2022.808606)
Supplement: Supplementary file 4 [file Table_1.docx]

| Pool | Peptide | Sequence |
| --- | --- | --- |
| 1 | VL1-20 | DIQMTTQSPSSLSASVGDRVT |
|  | VL9-28 | SSLSNASVGDRVTITCKASQN |
|  | VL17-36 | DRVTYITCKASQNVGTNVAWY |
|  | VL25-44 | ASQNPVGTNVAWYQQKPGKAP |
|  | VL33-52 | VAWYSQQKPGKAPKALIYSAS |
|  | VL41-60 | GKAPYKALIYSASFLYSGVPY |
|  | VL49-68 | YSASGFLYSGVPYRFSGSGSG |
| 2 | VL57-76 | GVPYSRFSGSGSGTDFTLTIS |
|  | VL65-84 | SGSGATDFTLTISSLQPEDFA |
|  | VL73-92 | LTISNSLQPEDFATYYCQQYN |
|  | VL81-100 | EDFAQTYYCQQYNIYPLTFGQ |
|  | VL89-108 | QQYNRIYPLTFGQGTKVEIKR |
|  | VL97-116 | TFGQFGTKVEIKRTVAAPSVF |
|  | VH1-20 | EVQLLVESGGGLVQPGGSLRL |
| 3 | VH9-28 | GGLVVQPGGSLRLSCAASGYV |
|  | VH17-36 | SLRLWSCAASGYVFTDYGMNW |
|  | VH25-44 | SGYVGFTDYGMNWVRQAPGKG |
|  | VH33-52 | GMNWNVRQAPGKGLEWMGWIN |
|  | VH41-60 | PGKGYLEWMGWINTYIGEPIY |
|  | VH49-68 | GWINFTYIGEPIYADSVKGRF |
|  | VH57-76 | EPIYKADSVKGRFTFSLDTSK |
| 4 | VH65-84 | KGRFNTFSLDTSKSTAYLQMN |
|  | VH73-92 | DTSKASTAYLQMNSLRAEDTA |
|  | VH81-100 | LQMNYSLRAEDTAVYYCARGY |
|  | VH89-108 | EDTAWVYYCARGYRSYAMDYW |
|  | VH97-116 | ARGYVRSYAMDYWGQGTLVTV |
|  | VH105-124 | MDYWPGQGTLVTVSSASTKGP |
|  | VH113-132 | LVTVSSSASTKGPSVFPLAPS |

Table S1 : sequences of the overlapping peptides and composition of the pools
